# Analysis of a Medical Education Instagram Account and Tips for Training Programs for Creating a Board Review Instagram Account

**DOI:** 10.7759/cureus.92127

**Published:** 2025-09-12

**Authors:** Saira Butt

**Affiliations:** 1 Internal Medicine/Infectious Diseases, Indiana University School of Medicine, Indianapolis, USA

**Keywords:** board review, instagram, meded, medical education, social media

## Abstract

Social media has emerged as a powerful platform for modern medical education, yet best practices for training programs' use of it remain underdeveloped. With its visual format and high engagement rates among younger demographics, Instagram offers a unique opportunity to supplement traditional learning. This paper analyzes the performance of a medical education Instagram account managed by an academic Infectious Diseases division. We aim to provide a replicable framework for other training programs seeking to establish an educational social media presence for board review preparation. A strategic, dual-pronged approach that combines community-building content with high-yield educational material can create a highly effective educational tool. This article provides tips for training programs to develop their Instagram accounts, focusing on creating engaging, board-style clinical cases to enhance trainee learning.

## Editorial

The landscape of medical education is continually evolving, driven by the learning preferences of new generations of trainees. Millennial and Gen Z physicians, who have grown up with digital technology, increasingly seek educational resources that are accessible, visually engaging, and integrated into their daily lives [1]. Social media platforms are potent tools for knowledge dissemination, professional networking, and "just-in-time" learning. [2,3]

Instagram, a visually centric platform with over two billion users, is well-suited for medical education. Its format supports high-quality images, short videos (Reels), and interactive "Stories," making it ideal for presenting clinical findings, radiologic images, and bite-sized educational pearls [4]. While many institutions have a social media presence for marketing, the use of Instagram as a dedicated, curriculum-enhancing tool by residency or fellowship programs is an area ripe for exploration [5,6].

This paper analyzes an Instagram account from an academic Infectious Diseases (ID) division (https://medicine.iu.edu/internal-medicine/specialties/infectious-diseases). The division's fellowship is a two-year, Accreditation Council for Graduate Medical Education (ACGME)-accredited program that trains physicians in the diagnosis and management of a wide array of infectious diseases through diverse clinical rotations and offers specialized tracks, including in antimicrobial stewardship, infection prevention, medical education, and global health.

The fellowship program director manages the account and creates and posts the board review cases. Contributions are also made by other faculty and fellows, who provide images of announcements and community engagement. By examining its performance metrics and content strategy, we aim to achieve two goals: (1) Describe the key factors contributing to the audience engagement and educational outreach success, and (2) provide a practical, step-by-step blueprint for other training programs to create and maintain their educational accounts, specifically focusing on developing board review-style cases.

We conducted a retrospective, descriptive analysis of the medical education Instagram account ‘IUIDfellowship’. The data was collected from the platform’s native analytics suite, the Instagram professional dashboard, for the 90 days ending July 24, 2025. At the close of this period, the account had 1,053 followers. Key performance indicators (KPIs) were extracted, including audience reach (total views, accounts reached), audience engagement (total interactions, accounts engaged), and profile activity (profile visits, external link taps). Content performance was analyzed by type (post vs. story) and by sorting top posts based on views, interactions, and shares. The Instagram dashboard qualitatively analyzed the thematic content of the top-ranking posts.

**Figure 1:**
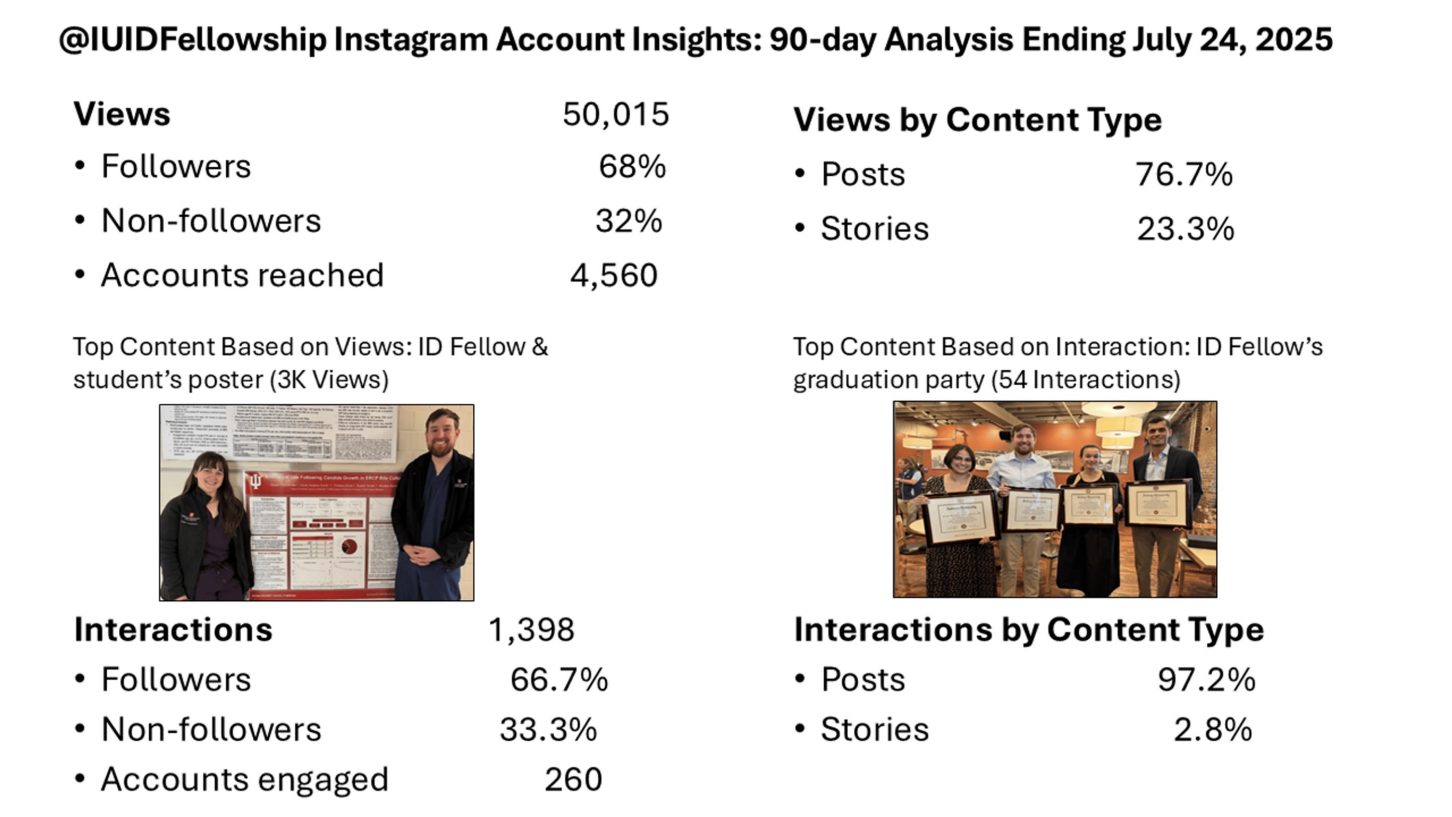
90-day Analysis of @IUIDFellowship Instagram Account

During the 90 days, the account generated 50,015 views, reaching 4,560 unique accounts (Figure 1).

Views were primarily driven by followers (68%) over non-followers (32%), indicating a loyal core audience. The account garnered 1,398 interactions from 260 unique accounts, with followers accounting for 66.7% of engagement. Analysis by content type revealed that posts were the primary driver of performance, accounting for 76.7% of views and 97.2% of interactions. In contrast, stories accounted for 23.2% of views and 2.8% of interactions. Over the same period, the account dashboard registered 491 profile visits and seven external link taps.

Analysis of follower activity showed peak engagement times on weekdays between 6:00 AM and 3:00 PM, providing a strategic window for posting. The qualitative analysis revealed two distinct, successful content pillars: (1) Community Building: Posts celebrating professional milestones (e.g., the "Chief Fellow 2025-2026" announcement, 53 interactions) and photos of faculty and fellows at events or in the hospital generated the highest number of direct interactions (likes and comments). This content was central to building community and personal connection with the program. (2) Educational Outreach: Posts with clear, didactic value received the most shares. The top-shared posts (six shares each) were educational infographics about foodborne bacteria, visual guides to tick identification, and a micrograph of Ehrlichia in a neutrophil. This indicates that while users interact with personal content, they share educational content, effectively amplifying the program's educational reach. Table 1 provides tips for other training programs to create a board review-focused Instagram account. 

**Table 1:** Tips for Training Programs for Creating a Board Review Instagram Account HIPAA: Health Insurance Portability and Accountability Act

Table 1. Tips for Training Programs for Creating a Board Review Instagram Account
Based on our analysis, we propose the following framework for creating a successful educational Instagram account focused on board review.
Phase 1: Foundation and Strategy
Start by defining the goals of the account. Is the primary goal trainee education, recruitment, or both? Define your target audience (e.g., future applicants, current trainees in other similar programs). Review and comply with the individual institution's guidelines on branding, social media policy, and HIPAA compliance. Designate 1-2 faculty members, 1-2 learner champions, and/or program coordinator to manage the account, distribute the workload, and ensure continuity. Plan to post consistently (e.g., 2-3 times per week) and can adjust based on review of analytics to identify peak follower times for optimal scheduling. The team can use an Excel or Google Docs sheet to plan posts in advance.
Phase 2: Content Creation - The "Case of the Day or Week" Model
The "Case of the Week" is an ideal format for board review on Instagram. Use a clinical image (e.g., a rash, a striking X-ray, a Gram stain), add the question stem with case presentation and relevant labs and imaging, comment and add the answer along with high yield "pearls" about the disease, diagnostics, and treatment, use bullet points for readability, and add references to the picture, question stem, and answer explanation.
Phase 3: Engagement and Growth. Include a mix of broad (#MedEd) and niche (#IDmeded, #InfectiousDiseases, #idBoardReview) hashtags. Use the "Poll" or "Quiz" sticker in Instagram Stories for quick-fire questions. Ideally, post the answer 24 hours later to drive daily engagement, or could post the answer right away. Encourage faculty and residents to share the posts on their professional accounts to broaden reach. Actively reply to comments to show that real people manage the account and that it fosters community.

This Instagram account's success stems from a strategic blend of content that serves two different but complementary purposes. Community-building posts foster a loyal audience, while educational posts establish the account as a credible, valuable resource.

The 90-day analysis of an infectious diseases fellowship program's Instagram account shows that board review-style cases are the primary drivers of content sharing, amplifying the program's reach and establishing its credibility as an educational resource. Community-building content, such as faculty highlights and fellow announcements, creates an interactive core audience. The paper provides tips for other academic programs seeking to leverage Instagram for medical education.

This is a single-account analysis over a limited period. The metrics do not directly measure learning outcomes or knowledge retention. However, the data on shares and engagement serve as strong surrogate markers for educational value and audience interest.
